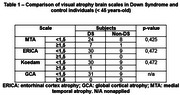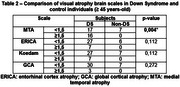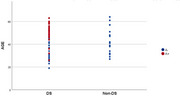# Amyloid Status and Cerebral Atrophy in Down Syndrome: a visual scale‐based analysis through normal and pathologic aging

**DOI:** 10.1002/alz70856_106085

**Published:** 2026-01-07

**Authors:** Isabela dos Santos Alves, Mateus Rozalem Aranha, Daniele de Paula Faria, Dimitri Brigide De Almeida Mantovani, Laura Cavalcanti de Oliveira, Claudia da Costa Leite, Douglas Mendes Nunes, Alexandre Bejanin, Carlos Alberto Buchpiguel, Juan Fortea, Artur Martins Coutinho

**Affiliations:** ^1^ Neuroradiology Section, Hospital Sírio‐Libanês, São Paulo, Brazil; ^2^ Center of Nuclear Medicine, Institute of Radiology, Hospital das Clínicas, Faculdade de Medicina da Universidade de São Paulo, São Paulo, São Paulo, Brazil; ^3^ Sant Pau Memory Unit, Hospital de la Santa Creu i Sant Pau, Institut de Recerca Sant Pau ‐ Universitat Autònoma de Barcelona, Barcelona, Spain; ^4^ Neuroradiology Section, Department of Radiology, Hospital de la Santa Creu i Sant Pau, Biomedical Research Institute Sant Pau, Universitat Autònoma de Barcelona, Spain, Barcelona, Spain; ^5^ Universidade de São Paulo, São Paulo, SP, Brazil; ^6^ University of São Paulo Medical School, São Paulo, São Paulo, Brazil; ^7^ University of São Paulo Medical School, São Paulo, Brazil; ^8^ LIM44, Hospital das Clinicas HCFMUSP, Faculdade de Medicina, Universidade de Sao Paulo, Sao Paulo, Sao Paulo, Brazil; ^9^ Hospital das Clinicas ‐ FMUSP, Sào Paulo, Brazil; ^10^ CIBERNED, Network Center for Biomedical Research in Neurodegenerative Diseases, National Institute of Health Carlos III, Madrid, Spain; ^11^ Sant Pau Memory Unit, Department of Neurology, Hospital de la Santa Creu i Sant Pau, Institut d'Investigació Biomèdica Sant Pau (IIB SANT PAU), Facultad de Medicina ‐ Universitat Autònoma de Barcelona, Barcelona, Spain; ^12^ Nuclear Medicine Section, Hospital Sírio‐Libanês, São Paulo, São Paulo, Brazil; ^13^ Barcelona Down Medical Center, Fundació Catalana Síndrome de Down, Barcelona, Spain

## Abstract

**Background:**

Down syndrome (DS) is considered a genetic form of Alzheimer's disease (AD) and is characterized by early neurodegeneration and cortical atrophy. Subjective scale‐based visual evaluation of brain atrophy is widely used in clinical neuroradiology. This study aims to evaluate brain atrophy scales in DS individuals from a young age and investigate its relationship with cortical amyloid status.

**Methods:**

A bi‐centric retrospective analysis was conducted at Hospital das Clínicas, Brazil, and Hospital de la Santa Creu i Sant Pau, Spain, using 3T Magnetic resonance imaging (MRI) data from individuals aged 18 years or older. Amyloid status was classified as positive (A+) or negative (A‐) based on [11C]PiB‐PET‐amyloid scans obtained within 30 days of MRI or cerebrospinal fluid analysis, with a positive result defined as an Aβ42/40 ratio < 0.062. MRI was independently analyzed by two expert neuroradiologists, who assigned scores for medial temporal atrophy (MTA), entorhinal cortex atrophy (ERICA), posterior cortical atrophy (Koedam scale), and global cerebral atrophy (GCA) for each hemisphere. The inter‐rater agreement was satisfactory, and the average (right‐left) from the more experienced neuroradiologist was used. Individuals were then separated into two groups (younger and older than 45).

**Results:**

106 individuals were initially evaluated, and 25 were excluded from the final analysis (total included N = 81). The mean age of participants was 42.7 years (19‐64 years). The sample consisted of 64 DS individuals (35% females) and 17 non‐DS controls (58% females). Thirty individuals were A+, all of them DS individuals. DS individuals exhibited higher atrophy scales, however, reaching statistical significance only for the MTA scale (hippocampal atrophy) in the ≥ 45‐year‐old group (tables 1 and 2); such atrophy was linked to higher A+ prevalence (*p*‐value < 0.05). Furthermore, higher A+ prevalence was observed in DS overall (*p*‐value < 0.05), including younger participants under 45 (Figure 1).

**Conclusions:**

The MTA scale detected early‐stage hippocampal atrophy in DS, which was also linked to amyloid deposition since early adulthood, highlighting the use of this scale as a proxy for AD pathology in DS.